# Fractional Frequency Reuse Scheme for Device to Device Communication Underlaying Cellular on Wireless Multimedia Sensor Networks

**DOI:** 10.3390/s18082661

**Published:** 2018-08-13

**Authors:** Jeehyeong Kim, Teasung Kim, Jaewon Noh, Sunghyun Cho

**Affiliations:** Department of Computer Science and Engineering, Hanyang University, 55 Hanyangdaehak-ro, Sangnok-gu, Ansan, Gyeonggi-do 426-791, Korea; manje111@gmail.com (J.K.); gtsk623@gmail.com (T.K.); wodnjs1451@gmail.com (J.N.)

**Keywords:** wireless mobile sensor network, fractional frequency reuse, D2D communication, interference management

## Abstract

Wireless multimedia sensor networks (WMSNs) have been improved with the increase of multimedia data. In WMSNs, a centralization problem can occur because of large-size multimedia data. It is necessary to consider device-to-device (D2D) communication. We focus on D2D WMSN based on cellular networks. Sensors in the D2D WMSN can non-orthogonally use a cellular link, which is a wireless communication channel between a sensor and an aggregator, and a D2D link, which is the channel between sensors. As a result, it has more complex interference environments than an ordinary system. Therefore, it is a key factor to manage the varying inter-cell interference effectively for throughput improvement. We propose an interference mitigation scheme that can be applied to D2D WMSN. In the proposed scheme, a cell is separated into six zones and orthogonal frequency is allocated to each zone for cellular links. The frequencies allocated to cellular links are reused by D2D links of neighboring zones. The simulation results show that the throughput of the proposed scheme increases two times compared to a static frequency allocation scheme.

## 1. Introduction

As enhancements of devices’ capabilities to record and play video continue to progress, demands to cope with the mobile multimedia data traffic also increase [1,2,3]. The demands have affected not only the cellular networks, but also sensor networks. Wireless multimedia sensor networks (WMSNs), wireless sensor networks (WSNs) for targeting the multimedia data, have been developed [4]. A sensor that can collect multimedia data is called a multimedia sensor in WMSN. The multimedia sensor can be used in various areas such as transmitting multimedia, checking traffic, and monitoring nature, people, and machines [5,6]. The WMSN uses multimedia sensing technologies to monitor peripheral situations and sends collected data to an aggregator for reporting [7]. An application in WMSN may require several months of operating lifetime [8]. Since WMSN consists of sensors, the WMSN technologies have been developed to ensure Quality of Service (QoS) and to overcome limited computing power and energy of sensors [4,5,9,10]. As these problems of limitation are reduced, the WMSN can be applied to future smart cities, security systems, or industries [11]. In WMSN, it is important to communicate efficiently and to achieve high throughput because large multimedia data are transmitted such as high resolution image, audio, and video stream [12]. Therefore, reliable and efficient wireless communication should be offered between sensor and data aggregator [13,14].

Many problems are considered that can occur in WMSN such as deployment costs, energy-efficient routing, network coverage, and connectivity [15,16,17]. One of the fundamental deficiencies that cause these problems is a centralized structure. All generated data were finally sent to a central aggregator (CA) in previous studies. Unlike the typical WSN system, the amount of traffic is very large in WMSN. It is a significant burden for the CA, and it can degrade the overall performances of WMSN. Thus, device-to-device ( D2D) communication should be considered in WMSN. The D2D communication in WMSN can be used for various services such as live streaming services and mobile aggregating systems. For the case of the streaming service, local streaming broadcast to personal devices in a stadium can be considered. The data can be broadcast to local networks. To reduce the dense overhead to the central system, the traffic should be distributed as much as possible. In the services, D2D links of WMSN can be considered. Additionally, mobile aggregating is one of the candidates to use D2D links for WMSN, which means that sensor networks use a mobile device as an aggregator [18,19]. To support the mobile aggregator, multi-hop communication should be considered, where a D2D link is adopted.

**Figure 1:**
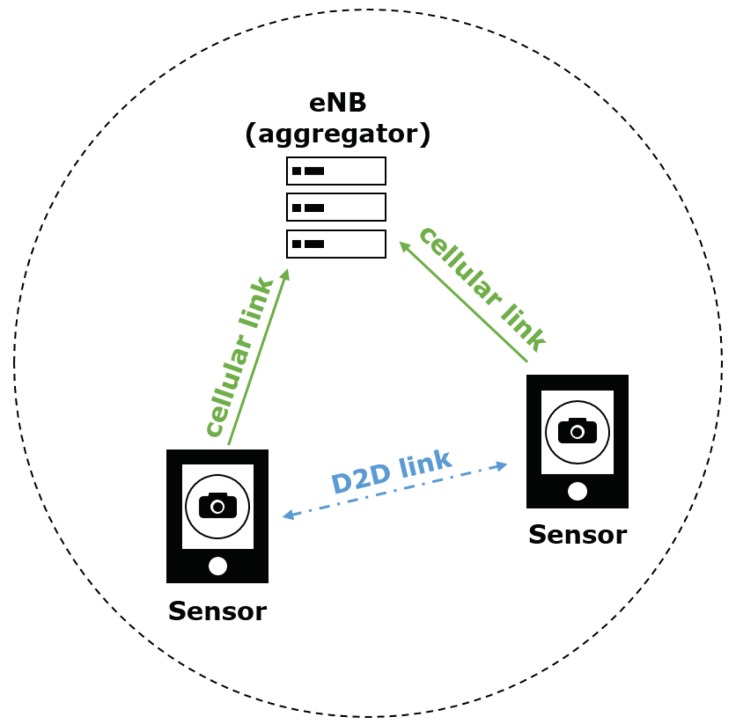
Cellular link and D2D link in WMSN.

We focus on a D2D WMSN with a cellular link and a D2D link as shown in Figure 1. There are two types of D2D links: overlaying and underlaying types [20,21]. The overlaying system is one in which a fraction of frequency resources is allocated to the D2D link. There is no interference between the uplink and D2D link, but bandwidth efficiency is degraded. Otherwise, the underlaying system is one in which a D2D link shares the same channel resources with the uplink. It means that the two links are based on a non-orthogonal system. The underlaying system can be regarded as a fault-tolerance mechanism such as [22,23], which maximizes performance while allowing some faults of links. Recently, studies of the D2D link underlaying system consider QoS requirements. The authors of [24,25] consider the minimum QoS requirement to avoid the dissatisfaction of the minimum SINR. In addition, the authors of [26] propose a radio resource allocation scheme that considers the intra cell interference and increases the throughput of whole cell. The study in [27] covers the various uplink resource allocation algorithms for the optimal allocation of the subcarriers and transmission powers in the SC-FDMA cellular networks. In [28], a bit allocation scheme is introduced to minimize the power consumption and avoid the interference between subcarriers so that the scheme can guarantee the QoS of each user.

According to the studies above, the key factor of the D2D system is an interference management. We propose an improved FFR scheme to gain high throughput and to handle both of the interferences. In the proposed scheme, a cell is separated into six equal portions called zones and the given radio frequency is also divided into six fractional pieces. The proposed scheme allocates each orthogonal frequency to cellular links of six zones one by one. In addition, each D2D link uses resources that do not overlap with the cellular link of the zone. Therefore, interference between the two links can be reduced by the proposed scheme. The contribution of this paper is as follows:

- We propose a novel interference management scheme by dividing cells into six zones for D2D links.
- The efficiency of proposed scheme is mathematically described by a geographical average distance.
- We implement the proposed scheme on a computer simulation to show various aspects of uplinks and D2D links.

This paper is organized as follows. Section 2 describes the scenarios in which the proposed network can be applied. Section 3 explains the proposed FFR scheme. Section 4 describes performance evaluation results. The conclusion will be discussed in Section 5 and future work is represented in Section 6.

## 2. Related Works

Various studies about conventional resource allocation for WSN are introduced in [29]. Thus, we describe several extensional studies using FFR for resource allocation. Specifically, the studies applying FFR for D2D communication are described as separately.

### 2.1. Resource Allocation Based on the Fractional Frequency Reuse

Most studies of fractional frequency reuse are based on an inter-cell interference coordination scheme (ICIC) [30], where all frequencies are available in the central region of a cell and partitioned frequencies are allocated for cell-edge areas. It reduces inter-cell interference because cell border regions use orthogonal frequency to uplink. In [31], a directional FFR (D-FFR) is considered, where a cell is divided into three sectors based on the ICIC scheme. From optimal configuration, the study shows that the D-FFR increases 60% of the throughput capacity relative to the omnidirectional-FFR scheme. In [32], the authors adopt FFR with multiple-input-multiple-output (MIMO) and heterogeneous networks (Hetnets). The authors in [33] propose a resource allocation with FFR for Hetnets and LTE Femtocell systems, which employs very high number sectors in a cell. In the [34], a buffered FFR scheme is proposed for LTE-Advanced Hetnets. It also adopts a more complex cell partitioning scheme. The authors in [35] propose an automata-based FFR scheme to allow a self-organized network to emerge. As shown in the above studies, FFR is still considered for various wireless systems to management resources.

### 2.2. D2D Communication Underlaying Uplink Based on the Fractional Frequency Reuse

There are many studies of D2D communication underlaying uplink systems based on cellular or WSNs [36]. In particular, we introduce studies based on FFR as related works. There is D2D communication with FFR based on ICIC introduced in [37,38]. In [39], the coverage performances of both links are mainly analyzed with a Poisson point process (PPP) model with a similar system. In [40], an additional fractional power control scheme to the D2D communication on the FFR-based system is provided. The authors in [41] also propose FFR for D2D communication underlaying cellular systems. It separates a cell into three inner regions and three outer regions to achieve higher throughput. In [42], LTE-A is considered with a D2D link on the FFR system. In [43], a dynamic power control scheme is proposed for the same system. These studies describe how it is convincing to apply D2D communication on the FFR-based systems.

### 2.3. High Sectored Cells on the Fractional Frequency Reuse

The high-sectored cells scheme, which consists of more than six sectors, is not a new concept in wireless communication. In [44], a sector offset configuration scheme is introduced. Each base station has two sector configuration, and a sector configuration consists of three hexa-cells. The pattern of second configuration is shifted by 60 degrees to implement the six-sectored cell. In [45], higher order sectorization gains are evaluated, with 6, 9, 12 and 15 sectored cell sites. For the D2D links, there are several six-sectored studies. The authors of [46] propose a D2D-enabled Hetnet system based on a six-sector system. It also adopts On/Off switching frequency to reduce the number of outage users. In [47], the authors propose a virtual sectoring concept to sectoring based on D2D communications. Because of the virtual sectoring, the sectoring can be controlled adaptively according to a density of D2D pairs. According to the above studies, FFR with a high-sectored cells scheme is still able to be considered. Simultaneously, it implies that the scheme is sufficiently implementable. Based on the related works, we propose a resource allocation with six-sectored cells for uplink and D2D link.

## 3. Proposed Scheme

### 3.1. System Model

**Figure 2:**
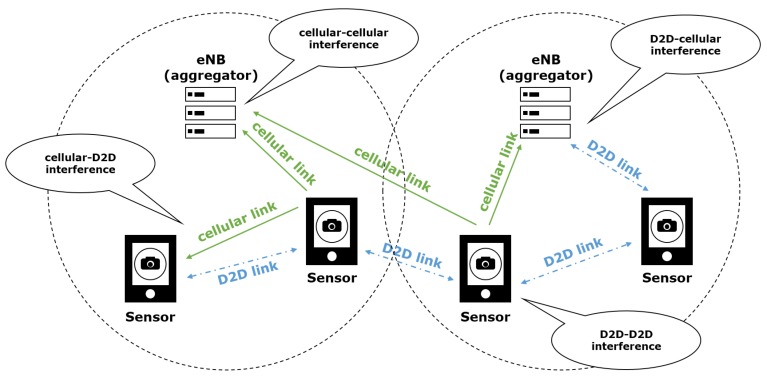
The case of interference in D2D WMSN.

In the sensor deployed systems considered, the cellular channel is divided into cellular and D2D links. A sensor can be connected to an aggregator via a cellular link or connected to another sensor via a D2D link. Because of these links, there are four interference cases as shown in Figure 2. The first two cases are the interference between cellular links and between D2D links. This interference can occur in both intra-cell interference and inter-cell interference in the case that the same frequency is allocated to the same type of link. Another case is the interference between cellular and D2D link. The process for sensors to control the complex interference situation is a challenging issue. The proposed solution in this paper focuses on minimizing interference while maximizing throughput by allocating the given radio frequency intelligently to each link.

### 3.2. Proposed FFR-3 Scheme

**Figure 3:**
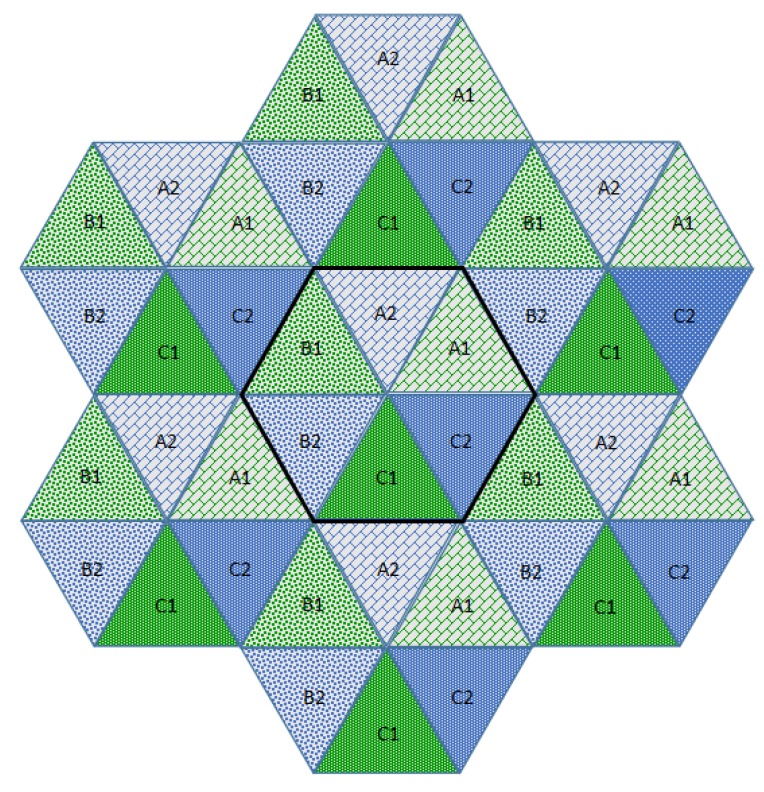
The cell structure in FFR-3.

**Figure 4:**
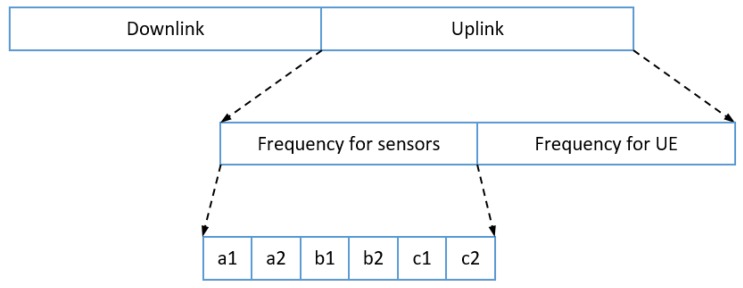
Frequency partitioning in FFR-3.

**Figure 5:**
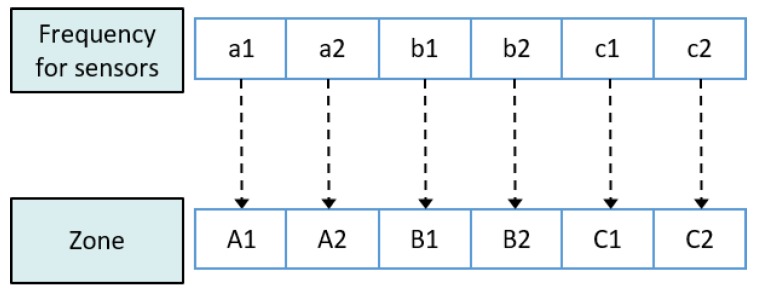
Frequency allocation to cellular links in FFR-3.

**Figure 6:**
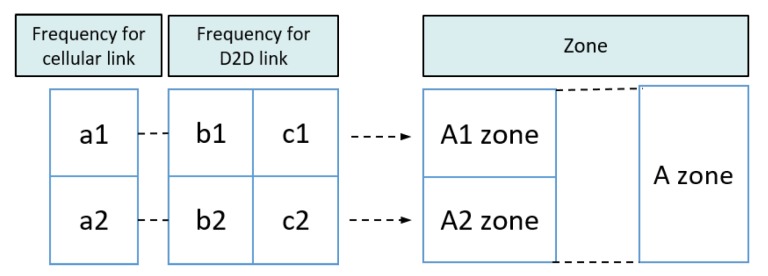
Frequency allocation to cellular link and D2D link.

In this paper, we propose a resource allocation scheme called fractional frequency reuse 3 (FFR-3). The reason for naming the proposed scheme FFR-3 is that interference mitigation and resource reuse methods are designed by dividing each cell and radio frequency into three areas. In the semi-static scheme, some resources are fixedly allocated for particular links and some resources are reused across links. In the proposed FFR-3, fixed orthogonal resources are allocated to cellular links in the same cell to avoid mutual interference. On the other hand, to minimize throughput loss, D2D links reuse resources allocated to cellular links to the extent that interference is minimized. The detailed procedure of the proposed scheme is as follows. Figure 3 and Figure 4 show the cell structure and frequency partitioning in FFR-3, respectively. As shown in Figure 3, a cell is divided into three sectors named *A*, *B*, and *C*. Each sector is again divided into two zones, and each zone is identified by a number after the sector name such as A1 and A2. As a result, each cell is divided into six zones consisting of A1, A2, B1, B2, C1 and C2. In FFR-3, uplink resources are divided into frequencies for UEs and for sensors. The frequency for sensors is divided into six sections as shown in Figure 4. The proposed FFR-3 allocates orthogonal resources to cellular links in the same cell. As seen in Figure 5, orthogonal frequencies consisting of a1, a2, b1, b2, c1 and c2 are allocated to cellular links of a zone from A1 to C2, exclusively. Accordingly, there is no intra-cell interference for cellular links. Moreover, the inter-cell interference is also avoided in FFR-3 by designing cell structure so that zones of the same name are not contiguous. To avoid the interference between cellular and D2D links and increase the cell throughput, the D2D links in each zone reuse the frequencies used by the cellular links in the neighboring zones. For example, as seen in Figure 6, the D2D links in A1 zone use b1 and c1 frequencies that are used by cellular links in B1 and C1 zones, respectively. In the same way, the D2D links in A2 zone uses frequencies b2 and c2 used by the cellular links in the neighboring zones. According to the cell structure of FFR-3, the D2D links of each zone allocate orthogonal resources to the D2D links of the neighboring zone. As a result, in FFR-3, each zone has one frequency section for cellular links and two frequency sections for D2D links. Note that frequency reuse factor (FRF) for the cellular link is one because one zone, which is 1/6 of the entire cell area, uses 1/6 of the entire frequency domain. FRF for the D2D link is two because one zone, which is 1/6 of the entire cell area, uses 2/6 of the entire frequency domain.

**Figure 7:**
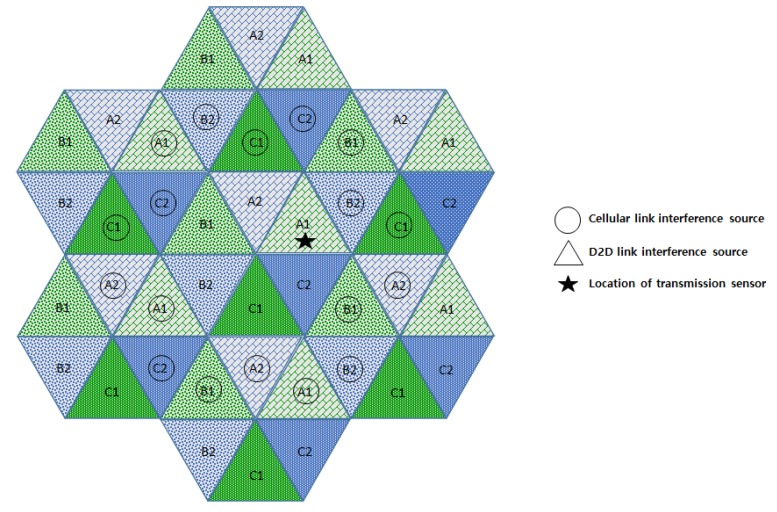
Interference sources to the target cellular link in a semi-static scheme.

**Figure 8:**
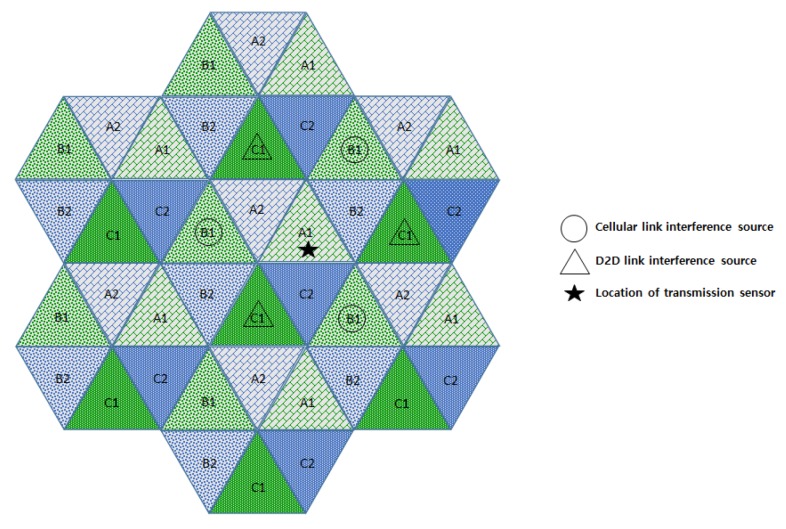
Interference sources to the target cellular link in FFR-3.

In this section, we compare the interference effects of the proposed FFR-3 with the LTE D2D system, which has a similar interference environment to the sensor deployed system. The resource allocation in the comparative LTE D2D system assumes the semi-static scheme in [48], which is orthogonal resource allocation to uplink and D2D link. Figure 7 and Figure 8 show the interferers affecting the target cellular link in semi-static and FFR-3, respectively. As shown in Figure 7, there is no intra-cell interference for the cellular link in the existing semi-static scheme. This is because orthogonal resources are allocated between D2D and cellular links in the same cell and time domain interference control is applied between cellular links. On the other hand, in the proposed FFR-3, there is intra-cell interference from the D2D link to the cellular link. This is because the D2D link reuses the cellular link resources of the adjacent zones to minimize the throughput reduction in FFR-3. However, since the D2D link basically uses a smaller transmission power than the cellular link, the interference effect on the cellular link is relatively small. However, in the existing semi-static scheme, the inter-cell interference effect on cellular link is much higher than FFR-3. As shown in Figure 7 and Figure 8, there are 18 neighboring zones interferers for the target cellular link in the semi-static scheme, while 11 neighboring zones interferers exist in FFR-3. As a result, an experienced interference is reduced because the number of interferers the adjacent zones is reduced. It is described mathematically in Section 3.4.

**Figure 9:**
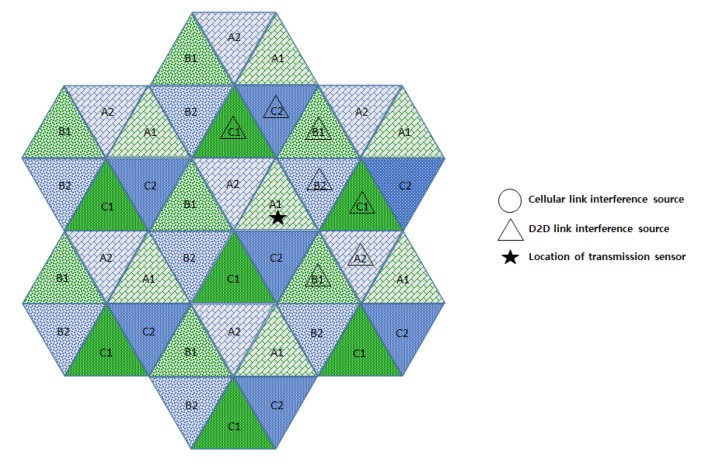
Interference sources to the target D2D link in a semi-static scheme.

**Figure 10:**
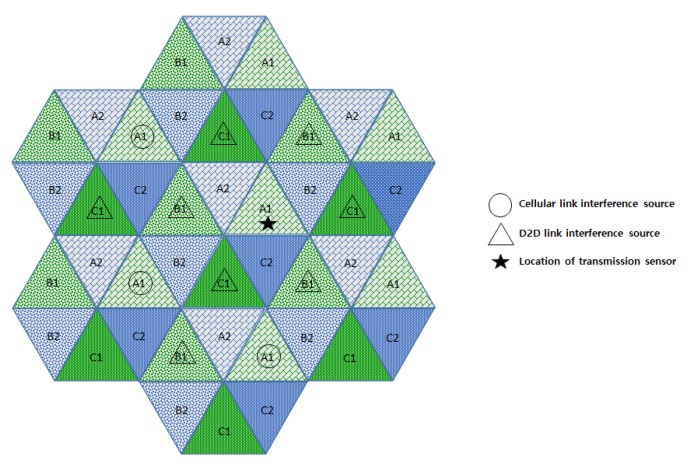
Interference sources to the target D2D link in an FFR-3 scheme.

Figure 9 and Figure 10 show the interferers affecting the target D2D link in semi-static and FFR-3, respectively. D2D link interference has characteristics similar to cellular link interference in FFR-3 and semi-static schemes. In the case of FFR-3, there is intra-cell interference to the D2D link. However, it is the interference from other D2D links, so the influence of interference is small due to small transmission power of D2D links. In addition, the six zone recoloring method significantly reduces the number of inter-cell interference sources. On the other hand, in the case of a semi-static scheme, there is no intra-cell interference for D2D links, but inter-cell interference exists. It means that the number of interferers is relatively large compared to FFR-3. Additionally, FFR-3 uses double frequency for the D2D links compared to semi-statics. In conclusion, FFR-3 enhances throughputs of D2D links with these two features: arranging locations of interferers and double frequency allocation. In Section 3.4, this is described in detail with geographical and mathematical analysis. In addition, in Section 4, various simulation results of the interference effects of FFR-3 and semi-static scheme are described.

### 3.3. Mathematical Modeling

In the semi-static scheme, it is inevitable to use the same frequency sections in neighboring zones. Thus, it requires an inter-zone interference manage scheme. In the proposed FFR-3 scheme, interference is mitigated without throughput loss by appropriately combining orthogonal resource allocation and resource reuse scheme for each zone. FFR-3 obtains more FRF gain compared to the typical semi-static scheme. As described in the previous section, in FFR-3, FRF of cellular link and D2D link are one and two, respectively. It means that the proposed FFR-3 is superior to the conventional scheme in terms of interference mitigation but also throughput gain.

In FFR-3, orthogonal frequencies are allocated to each zone in the same cell. However, there may be inter-cell interference from other cellular links in the adjacent cells, but the number of interferers is reduced and the distance from the interferer is increased. Therefore, we define SINR of FFR-3 as $SINR_{ffr,CE}^{sensor_{k}\left(Z_{An}\right)-eNB_{c}}$ where sensor *k* is located in zone $Z_{An}$, and it is connected with $eNB_{c}$ using cellular link. It can be derived with Table 1 as

(1) $$\begin{array}{cc} SINR_{ffr,CE}^{sensor_{k}\left(Z_{An}\right)-eNB_{c}} & =\frac{P_{t,b}G_{kc}}{IF_{An}+\left(IF_{Bn}+IF_{Cn}\right)+N_{0}}\\ & =\frac{P_{t,b}G_{kc}}{\sum_{i\in (Z_{An}\cap K_{CE}\cap {C_{c}}^{c})}P_{t,b}G_{ic}+\sum_{i\in (\left(Z_{Bn}\cup Z_{Cn}\right)\cap K_{D2D})}P_{t,s}G_{ic}+N_{0}}, \end{array}$$

where the interferers are classified into three groups, $IF_{An}$, $IF_{Bn}$, and $IF_{Cn}$. Each group means that interference from each zone. $IF_{An}$ is interference from the devices in all of zone An, the same zone with the transferring device. $IF_{An}$ can be described as $\left(Z_{An}\cap K_{CE}\cap {C_{c}}^{c}\right)$. It means that the interferers are in every zone $Z_{An}$, where n∈{1,2}. In addition, the interferers use the cellular link so they are elements of the set $K_{CE}$ and they are not included in the cell of $eNB_{c}$, which is $C_{c}$. $\left(IF_{Bn}+IF_{Cn}\right)$ describes interference from the the other zones, which is $\left(\left(Z_{Bn}\cup Z_{Cn}\right)\cap K_{D2D}\right)$. It is a set of interferers using D2D links, $K_{D2D}$ and is a union set of $Z_{Bn}$ and $Z_{Cn}$, where n∈{1,2}.

For a D2D link, two frequency sections are used. For example, the frequency sections, bn and cn are used as D2D links in the zone An. With the bn case, it is also used as a cellular link in Bn and used as a D2D link in Cn. It is the same as the case of cn in the zone An symmetrically. The SINR for the frequency section bn, $SINR_{ffr,D2D_{1}}^{sensor_{k}\left(Z_{An}\right)-sensor_{j}}$ is as follows:

(2) $$\begin{array}{cc} SINR_{ffr,D2D_{1}}^{sensor_{k}\left(Z_{An}\right)-sensor_{j}} & =\frac{P_{t,s}G_{kj}}{IF_{An}+IF_{Bn}+IF_{Cn}+N_{0}}\\ & =\frac{P_{t,s}G_{kj}}{\sum_{i\in (Z_{An}\cap K_{D2D}\cap {C_{j}}^{c})}P_{t,s}G_{ij}+\sum_{i\in (Z_{Bn}\cap K_{CE})}P_{t,b}G_{ij}+\sum_{i\in (Z_{Cn}\cap K_{D2D})}P_{t,s}G_{ij}+N_{0}}, \end{array}$$

where the $IF_{An}$ case means interference from the same zone of the other cells. $IF_{Bn}$ and $IF_{Cn}$ mean interference from all other zones. One of them is from the D2D link and the other is from the cellular link. It is changed in the case of cn, which is $SINR_{ffr,D2D_{2}}^{sensor_{k}\left(Z_{An}\right)-sensor_{j}}$. The order is not important because the two cases are symmetric. Thus, $SINR_{ffr,D2D_{2}}^{sensor_{k}\left(Z_{An}\right)-sensor_{j}}$ is given by

(3) $$\begin{array}{cc} SINR_{ffr,D2D_{2}}^{sensor_{k}\left(Z_{An}\right)-sensor_{j}} & =\frac{P_{t,s}G_{kj}}{IF_{An}+IF_{Bn}+IF_{Cn}+N_{0}}\\ & =\frac{P_{t,s}G_{kj}}{\sum_{i\in (Z_{An}\cap K_{D2D}\cap {C_{j}}^{c})}P_{t,s}G_{ij}+\sum_{i\in (Z_{Bn}\cap K_{D2D})}P_{t,s}G_{ij}+\sum_{i\in (Z_{Cn}\cap K_{CE})}P_{t,b}G_{ij}+N_{0}}, \end{array}$$

where n∈{1,2}.

In the semi-static scheme, there are inter-cell interferences from adjacent cells for both of the links, cellular and D2D links, but intra-cell interference does not exist. Therefore, the SINR of the two links, $SINR_{ss,CE}^{sensor_{k}-eNB_{j}}$ and $SINR_{ss,D2D}^{sensor_{k}-sensor_{j}}$ can be defined respectively as:

(4) $$SINR_{ss,CE}^{sensor_{k}-eNB_{j}}=\frac{P_{t,b}G_{kj}}{\sum_{i\in (K_{CE}\cap {C_{j}}^{c})}P_{t,b}G_{ij}+N_{0}},$$

(5) $$SINR_{ss,D2D}^{sensor_{k}-sensor_{j}}=\frac{P_{t,s}G_{kj}}{\sum_{i\in (K_{D2D}\cap {C_{j}}^{c})}P_{t,s}G_{ij}+N_{0}}.$$

### 3.4. Numerical Analysis

In the proposed method, deployments of nodes are managed per zone. In that case, the average distances between neighboring zones are required because average distance between neighboring zones are different according to type of neighboring zones. There are three types to neighbor interferences between zones for each link, respectively. For cellular link, we define $\tau_{A}$, $\tau_{B}$ and $\tau_{C}$ types of neighboring interference as shown in Table 2. In the cellular link, interference to the uplink is experienced at eNB, the center of a cell. Thus, estimating interference from neighboring zones is regarded as estimating distance between a point and a triangle geometrically. The average distance according to the types can be defined as follows:

(6) $$\begin{array}{c} \tau_{q}=D_{avg}\left(q\right)=\frac{\int_{q}rdS}{S}, \end{array}$$

where r is the distance between the point and the zone, and *q* means types of neighboring zones, $q\in \tau ,\tau =\{\tau_{A},\tau_{B},\tau_{C},\tau_{D},\tau_{E},\tau_{F}\}$. *S* is the area of a zone, and r is the distance between from a sensor to the eNB. The figures in Table 2 describe sensors in a zone and an eNB for the zone. The colored area means that area of interferers and the eNB is the target eNB, which experiences interference from the colored area. The case of the $\tau_{A}$ is that interferences come from zones in the same cell. It is an average distance from triangle to a vertex of the triangle, so $\tau_{A}$ is 0.607, assuming the length of one side of a zone is 1. Similarly, $\tau_{B}$, and $\tau_{C}$ are 1.175 and 1.541, respectively, and the formulations of them are described in Appendix A. For D2D links, there are another three types, $\tau_{D}$, $\tau_{E}$ and $\tau_{F}$, to describe interference from neighboring zones in Table 2. The distance between two zones can be derived from [49]. We adopt the power law propagation model in [50]. It models path-loss for both of cellular and D2D links. Thus, the received power is modeled as follows:

(7) $$\begin{array}{c} P_{r,m}=P_{t,m}hr^{-\alpha }, \end{array}$$

where *m* is the link whether cellular or D2D link, m∈b,s. *r* is the distance between the transmitter and receiver. α is the path-loss coefficient where α>2. *h* is the channel gain. In this modeling, the channel gain is considered as 1 to clarify the effect of distance on results, while it is considered as an exponentially distributed random variable in simulations in Section 4. The distance is redefined as a coefficient of the sector as $\tau_{q}$, where *q* is the type indicator of the tau in the Table 2. Thus, Equation (7) is redefined as

(8) $$\begin{array}{cc} P_{r,m,q} & =P_{t,m}h{\left(\tau_{q}r\right)}^{-\alpha }\\ & =P_{t,m}hr^{-\alpha }\tau_{q}^{-\alpha }. \end{array}$$

Experienced interferences at a node are from cellular and D2D links simultaneously. Thus, the $I_{CE,ffr}$ is

(9) $$\begin{array}{c} I_{CE,ffr}=\sum_{s\in N_{z}}\sum_{m\in \{CE,D2D\}}\sum_{q\in \tau }P_{r,m,q}u_{s,m}, \end{array}$$

where $u_{s,m}$ is the number of average users in a zone *s*, who use the link, *m*. We assume that users are deployed uniformly, so the $u_{s,m}$ is the same for every zone. $N_{z}$ is a set of neighboring zones of the zone *z*. In the ffr mode, channels are determined for each zone, and it is described in Figure 7. The number of neighboring zones which interfere with the *z* zone is denoted as $n_{source,destination,mode,zone}$, as shown in Table 3. The source is the link that the interferer use. The mode is the system they use, which consists of the ffr and the ss, which is the semi-static mode. Thus, it is simplified to determine zones of interfering sources by using *n*. Accordingly, Equation (9) is rederived as

(10) $$\begin{array}{cc} I_{CE,ffr} & =\sum_{q\in \tau }\left(u_{s,CE}n_{CE,CE,ffr,q}P_{r,CE,q}+u_{s,D2D}n_{D2D,CE,ffr,q}P_{r,D2D,q}\right)\\ & =\sum_{q\in \tau }\left(u_{s,CE}n_{CE,CE,ffr,q}P_{t,CE}hr^{-\alpha }\tau_{q}^{-\alpha }+\nu u_{s,CE}n_{D2D,CE,ffr,q}P_{t,D2D}hr^{-\alpha }\tau_{q}^{-\alpha }\right), \end{array}$$

where $u_{s,D2D}=\nu u_{s,CE}$. After arranging the common coefficients and constants related with the ffr design, Equation (10) is derived again as follows:

(11) $$\begin{array}{c} I_{CE,ffr}=\ell P_{t}\Psi_{CE,ffr}1, \end{array}$$

where $\ell =u_{s,CE}hr^{-\alpha }$, 1=[1,1,1], and $P_{t}=\left[P_{t,CE},\nu P_{t,D2D}\right]$. In addition, $\Psi_{CE,ffr}$ is denoted as

(12) $$\begin{array}{c} \Psi_{CE,ffr}=\left[\begin{array}{ccc} n_{D2D,CE,ffr,\tau_{A}}\tau_{A}^{-\alpha } & n_{D2D,CE,ffr,\tau_{B}}\tau_{B}^{-\alpha } & n_{D2D,CE,ffr,\tau_{C}}\tau_{C}^{-\alpha }\\ n_{CE,CE,ffr,\tau_{D}}\tau_{D}^{-\alpha } & n_{CE,CE,ffr,\tau_{E}}\tau_{E}^{-\alpha } & n_{CE,CE,ffr,\tau_{F}}\tau_{F}^{-\alpha } \end{array}\right]. \end{array}$$

Accordingly, Equation (1) can be derived with adopting Equation (11) as

(13) $$\begin{array}{cc} SINR_{ffr,CE} & =\frac{P_{r,CE}}{I_{CE,ffr}+I_{sh,ffr}+N_{0}} \end{array}$$

(14) $$\begin{array}{c} \;=\frac{P_{r,CE}}{\ell P_{t}\left(\Psi_{CE,ffr}+\Psi_{D2D,ffr}\right)1+N_{0}}. \end{array}$$

To simplify, $\ell P_{t}\left(\Psi_{CE,ffr}+\Psi_{D2D,ffr}\right)1$ can be replaced by $\gamma_{ce}^{ffr}P_{t,CE}+\delta_{D2D}^{ffr}P_{t,D2D}$ because others are constants excepts the power variables, $P_{t,CE}$ and $P_{t,D2D}$. Then, Equation (14) is redefined as

(15) $$\begin{array}{cc} SINR_{ffr,CE} & =\frac{P_{r,CE}}{\gamma_{CE,CE}^{ffr}P_{t,CE}+\delta_{D2D,CE}^{ffr}P_{t,D2D}}, \end{array}$$

(16) $$\begin{array}{cc} T_{ffr,CE} & =\frac{B}{6u_{z,CE}}\log_{2}\left(1+SINR_{ffr,CE}\right), \end{array}$$

where *T* is defined as throughput with the Shannon capacity. *B* is total system bandwidth per a cell. A zone uses $\frac{B}{6}$ of bandwidth because a cell has six zones. We assume that the bandwidth is allocated equally and sequentially to the users in the zone. It means that the same portion of the bandwidth is allocated to each user. Similarly, throughput for D2D link in ffr mode also can be derived as

(17) $$\begin{array}{cc} T_{ffr,D2D} & =\frac{2B}{6u_{z,D2D}}\log_{2}\left(1+SINR_{ffr,D2D}\right), \end{array}$$

where D2D link in ffr mode uses double bandwidth because the two frequency domains which are allocated for cellular are used as the D2D link in the other two zones. On the other hand, in the semi-static method, system bandwidth is divided into cellular and D2D links. We assume that it is separated with considering ratio of the number of users for each link. Therefore, $T_{ss,CE}$ and $T_{ss,D2D}$ are derived as

(18) $$\begin{array}{cc} T_{ss,CE} & =\frac{u_{z,CE}}{u_{z,CE}+u_{z,D2D}}\frac{B}{6u_{z,CE}}\log_{2}\left(1+SINR_{ss,CE}\right)\\ & =\frac{B}{6\nu (1+u_{z,CE})}\log_{2}\left(1+SINR_{ss,CE}\right), \end{array}$$

(19) $$\begin{array}{cc} T_{ss,D2D} & =\frac{u_{z,D2D}}{u_{z,CE}+u_{z,D2D}}\frac{B}{6u_{z,D2D}}\log_{2}\left(1+SINR_{ss,D2D}\right)\\ & =\frac{B}{6\nu (1+u_{z,CE})}\log_{2}\left(1+SINR_{ss,D2D}\right), \end{array}$$

where the bandwidth is allocated to a cell in semi-static mode. For instance, $6u_{z,CE}$ users use $\frac{u_{z,CE}B}{u_{z,CE}\;+\;u_{z,D2D}}$. Finally, we define FFR_GAIN to estimate performance enhancement of ffr mode compared to the semi-static mode as

(20) $$\begin{array}{c} FFR\_GAIN=\frac{T_{ffr,CE}+T_{ffr,D2D}}{T_{ss,CE}+T_{ss,D2D}}. \end{array}$$

**Figure 11:**
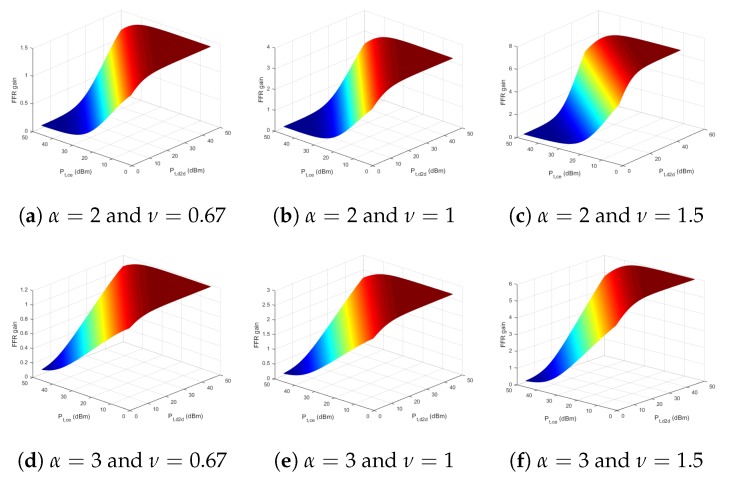
FFRgain versus $P_{t,CE}$ and $P_{t,D2D}$ with various α and ν.

Figure 11 shows the FFR_GAIN versus $P_{t,CE}$ and $P_{t,D2D}$ with various path loss exponents, α and the ratio of the number of users for each link, ν. We set 20 users for a zone, and the number of users for each link is determined according to ν. For instance, the ν=0.67 case means that 12 users use cellular link, and eight users use D2D link. The higher FFR_GAIN means that ffr mode is more appropriate than the semi-static mode in the case. The graphs have a common tendency: the FFR_GAIN is increased with high uses of D2D link. The graphs show that more $P_{t,D2D}$, the more FFR_GAIN in all cases. In addition, the FFR_GAIN is increased with higher ν comparatively. It implies that the more users using D2D links, the better performance of the ffr mode. According to the ffr design, D2D links use double frequency domains. It is the prime factor to enhance the FFR_GAIN. On the other hand, the FFR_GAIN is increased when the path loss exponent α is increased. If the α is high, the path loss would be increased, and the received power via longer distance is more reduced according to Equation (7). Then, the portion of cellular link in FFR_GAIN is decreased comparatively. As with the effects of $P_{t,D2D}$ as mentioned above, the more influence of the D2D link, the larger FFR_GAIN.

## 4. Performance Evaluation

### 4.1. Simulation Model

This section describes the performance evaluation results for the proposed scheme. We implement a simulation by Python. The simulation consists of three layers: node, cell, and data layer. The node layer defines a node class for configuration of each node. It manages the information of specific nodes, such as modes of the node, locations, powers, and channels including calculating experienced interference, SINR, and throughputs. The cell layer manages the nodes in perspective of cells. In the cell layer, distribution of nodes is controlled, and cell-throughput is calculated. Finally, the data layer collects the outputs from each distribution of nodes, and arranges the data to be easy to make graphs. The simulation environment is Python 3.6 on Anaconda 4.3.1, which is a mathematical library for Python. It operates on Window 10 Pro (Microsoft, Redmond, WA, USA). The system consists of i7-6700K CPU and 32 GB memory, but the simulation does not require this high-spec system.

Computational complexity of the framework is determined by the number of devices. Interference should be considered with only the devices in the same numbered zones. It means that when a device in zone A1 is the target device, A2, B2 and C2 devices are not necessary to be considered. Additionally, the devices in the same zone are also not necessary to be considered. However, they are comparatively small to estimate computational complexity. Therefore, the computational complexity of the proposed scheme can be described as $O(\frac{n(K_{CE}\cup K_{D2D})}{2})$. It is the same for both cellular and D2D links.

We compare the performance of the proposed scheme with the semi-static scheme, where each link has a dedicated frequency. The simulator for the performance evaluation assumes a three-tier cell environment. Table 4 shows the simulation parameters and values. The path loss model is the NLOS Winner II B5f model for urban areas [51,52]. The carrier frequency is assumed to 2 GHz. In the simulation, it is assumed that there are 2, 4 and 10 sensors in each zone. It means that there are 12, 24 and 60 sensors in a cell, respectively. In addition, we assume that 50% of sensors use the cellular link and the remaining 50% use the D2D link. Thus, the number of sensors that are connected to the cellular link per zone is 1, 2 and 5, respectively. Since one cell is composed of six zones, each cell has 6, 12 and 30 cellular links. Each experiment is conducted 1000 times and the results are averaged for the Monte Carlo method.

### 4.2. (Simulation Results) SINR and Throughput of the Cellular Links

**Figure 12:**
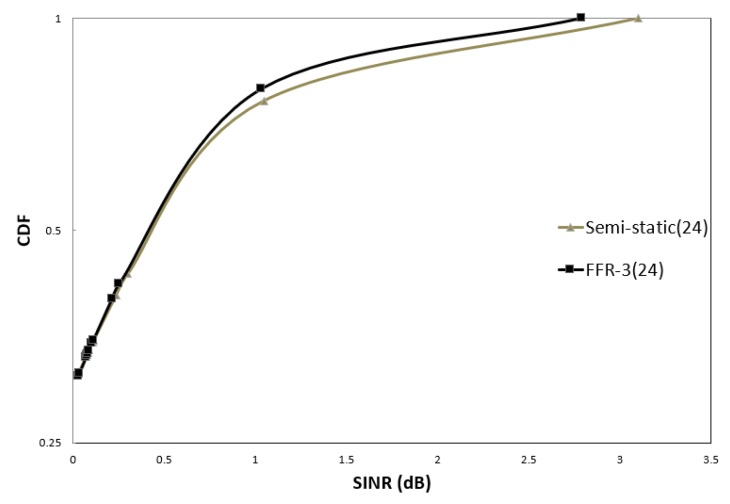
CDF of sensor SINR in cellular link (D2D Tx Power = 30 dBm).

**Figure 13:**
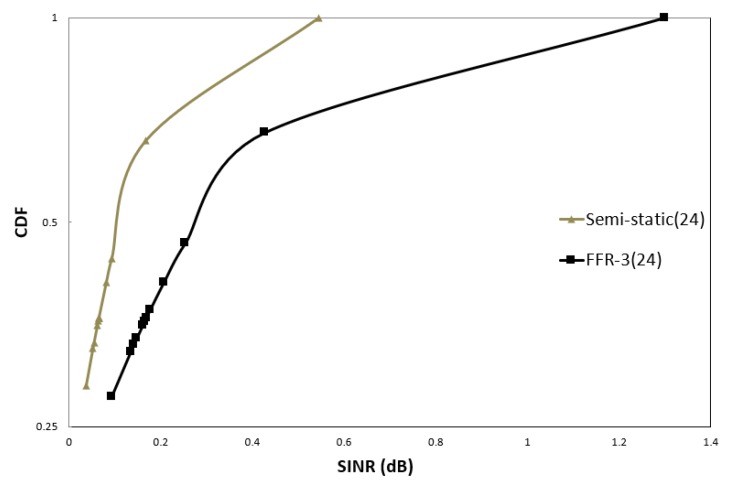
CDF of sensor SINR in cellular link (D2D Tx power = 25 dBm).

In order to evaluate the performance, SINR and throughput of cellular links and D2D links are compared in FFR-3 and semi-static scheme. Figure 12 and Figure 13 illustrate the CDF of the SINR of the cellular link for semi-static and FFR-3. In the legend of Figure 12 and Figure 13, the numbers in parentheses indicate the number of sensors per zone. Figure 12 compares SINR distributions of semi-static and FFR-3 when the transmission powers of cellular and D2D links are 30 dB and the number of sensors per zone is four. As shown in the results, the SINR performance of the cellular link in FFR-3 and the semi-static scheme is almost the same when the transmission power of the D2D and cellular link is the same. However, as shown in Figure 13, when the transmission power of the D2D link is less than that of the cellular link, FFR-3 has better SINR of the cellular link than the semi-static scheme.

**Figure 14:**
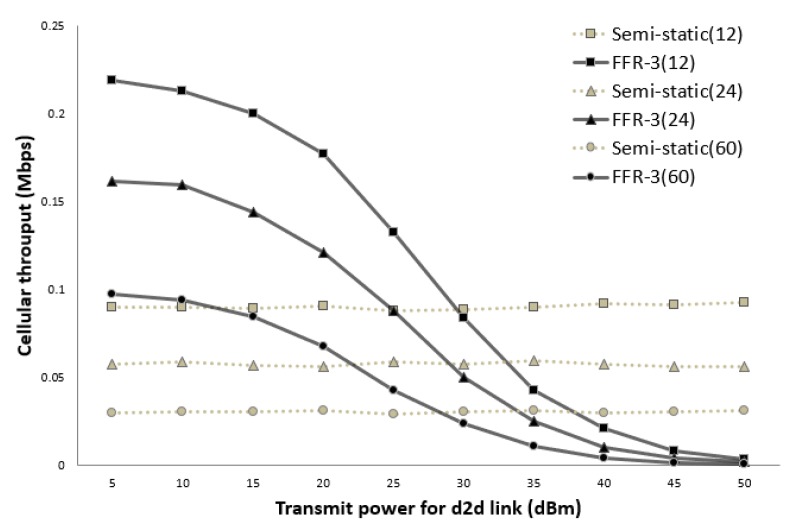
Cellular link throughput versus D2D transmit power (cellular Tx power = 30 dBm).

**Figure 15:**
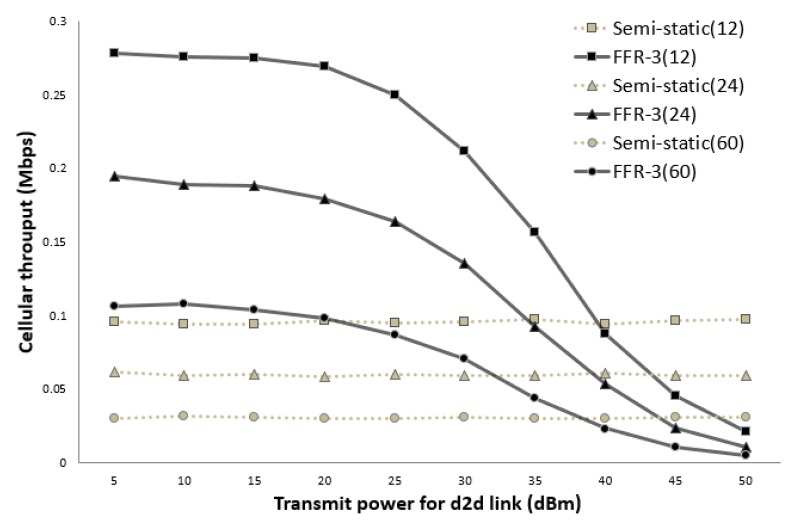
Cellular link throughput versus D2D transmit power (cellular Tx power = 40 dBm).

Figure 14 and Figure 15 show the average throughput of the cellular links according to the transmission (Tx) power of the D2D links when the cellular link Tx power is 30 dBm and 40 dBm, respectively. In the legend used in Figure 14 and Figure 15, the numbers in parentheses indicate the number of sensors per cell. In the semi-static scheme, the cellular link is affected by other cellular links in neighboring cells. Thus, the average throughput of the cellular links is constant even if the Tx power of the D2D links changes. On the other hand, the throughput of the cellular links in FFR-3 is affected by the Tx power of D2D links in neighboring cell. As shown in Figure 14 and Figure 15, when the transmission power of the D2D link is less than the transmission power of the cellular link, the throughput of the proposed algorithm is superior to that of the semi-static scheme. In the sensor deployed system, the D2D link is designed for short-range communication between sensors, and the cellular link is designed for communication between the sensor and the base station. Therefore, the transmission power of the D2D link is generally much less than the transmission power of the cellular link. This means that a spot in a real field would be on the left side of the graphs in Figure 14 and Figure 15. Thus, FFR-3 would be much better than semi-static in terms of the average throughput of the cellular links.

### 4.3. (Simulation Results) SINR and Throughput of the D2D Links

**Figure 16:**
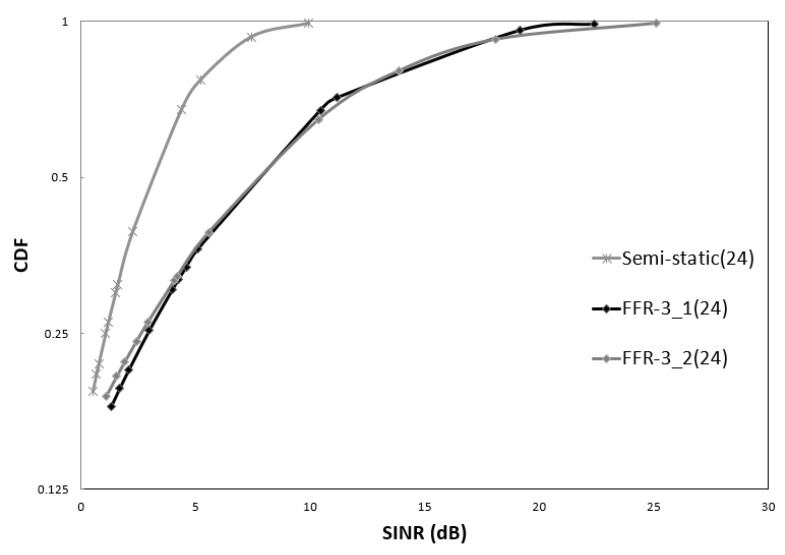
CDF of sensor SINR in D2D link (D2D Tx power = 30 dBm).

As described above, in FFR-3, two frequency sections are allocated for a D2D link in one zone. The two allocated frequencies are also symmetric in terms of geographical distribution, so they have similar SINR distributions. In Figure 16, FFR-3_1 and FFR-3_2 represent SINR distributions for the two frequency sections used by D2D links, respectively, and show similar distributions. Both frequency sections allocated for the D2D links in FFR-3 show superior SINR over the semi-static scheme.

**Figure 17:**
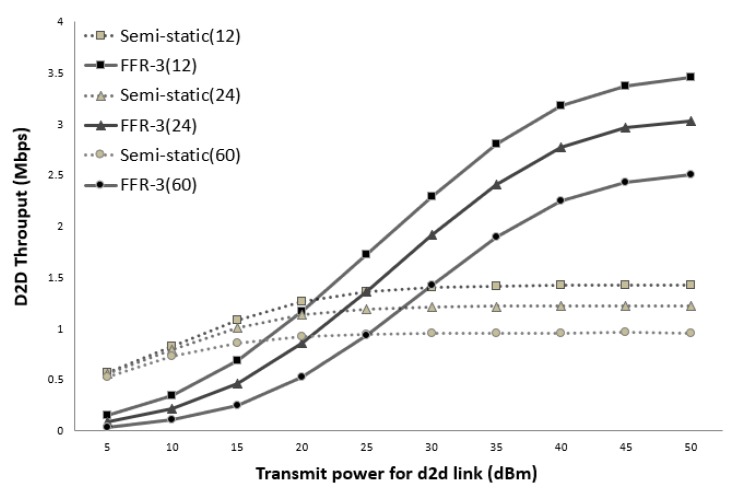
D2D link throughput versus D2D transmit power (cellular Tx power = 30 dBm).

**Figure 18:**
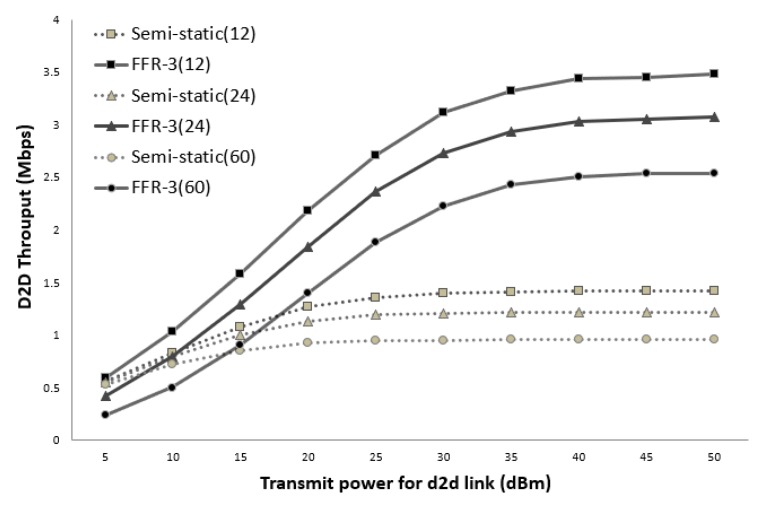
D2D link throughput versus D2D transmit power (cellular Tx power = 40 dBm).

Figure 17 and Figure 18 show the average throughput of the D2D links according to the transmission (Tx) power of the D2D links when the cellular link Tx power is 30 dBm and 40 dBm, respectively. As shown in the figure, the throughput of the D2D link in the semi-static scheme is hardly affected by the transmission power of the cellular link and other D2D links. This is because the target D2D link uses orthogonal resources with respect to the cellular link and the transmission power of all the D2D links is assumed to be the same. On the other hand, in FFR-3, the throughput of the D2D link is affected by the transmission power of the cellular link and the surrounding D2D link. This is because D2D links reuse the same radio frequency used by cellular links and D2D links in the adjacent zones. Thus, if the transmission power of the D2D link becomes larger than that of the cellular link, the throughput of the D2D link rapidly increases in FFR-3.

## 5. Discussion

If there is an optimal power allocation scheme, the performance of the proposed scheme would be much better. The proposed scheme is a complicated resource allocation scheme, which consists of D2D underlaying uplink and D2D, and it is managed by units of zone. According to numerical results and simulation results, the transmit power of D2D and cellular link is the most important factor to determine the performance of the proposed scheme. It means that the proposed scheme is sensitive with a power allocation scheme. If an optimal power allocation scheme is adopted on the proposed scheme, it can be better than the case of the static power allocation. Furthermore, the sectorization can be optimized. It can adopt a more higher faction algorithm in a cell if the smart antenna scheme can support it. Depending on the average transmit power for each link, the optimal fraction algorithm can also vary. To optimize the proposed scheme, the fraction algorithm and power allocation for each device should be optimized at the same time. This is future work of the proposed scheme.

## 6. Conclusions

In the WMSN, multimedia data should be transmitted in real time. It is essential to support high throughput for multimedia data from sensors. When all of data are transmitted to a central aggregator, a centralization problem can occur. Therefore, D2D communication should be considered in WMSN. In the D2D WMSN, using an underlaying system is more effective because sensors in an underlaying system can share a channel. Sensors can use channels as two links: the cellular link and D2D link. An inter-cell interference management scheme is required to use the same channel versus D2D and cellular links. Therefore, the required interference management scheme should be able to control the two links effectively. The proposed scheme, FFR-3, divides an ordinary cell into six zones and allocates an orthogonal frequency section for each zone. In addition, the frequency sections allocated cellular links are reused by the D2D links of the adjacent zones to increase throughput. Through this scheme, inter-cell interference is decreased and the FRF is increased to maximize throughput. It is expected that the throughput will be almost two times better than the semi-static scheme by controlling the transmission power of each link.

## Figures and Tables

**Table 1:** Definition of symbols.

Symbol	Definition
$K_{CE}$	A set of sensors using a cellular link
$K_{D2D}$	A set of sensors using a D2D link
$Z_{ai}$	A set of sensors in a zone which has sector *a* and number *i*
$C_{j}$	A set of sensors in a cell of eNB or sensor *j*
$P_{t,b}$	Tx power of a sensor for cellular link
$P_{t,s}$	Tx power of a sensor for D2D link
$P_{r,m,q}$	Rx power with mode *m*, m∈{CE,D2D} and neghboring type *q*
$N_{0}$	Noise power
$G_{kc}$	Channel gain between sensor *k* and eNB *c*
$G_{jl}$	Channel gain between sensor *j* and sensor *l*
$IF_{Xn}$	Interference from sensors in all zone Xn
$I_{l,m}$	Experienced interference at a node with using link *l* on mode *m*
$D_{avg}\left(q\right)$	Average distance between two zones with type *q*
$\tau_{q}$	Coefficient of average distance with type *q*, where $\tau_{q}\in \tau$
$u_{s,l}$	the number of average users in a zone *s* using the link *l*
μ	the ratio of the number of average users of using cellular case to using D2D case
*r*	distance between the transmitter and receiver
α	path-loss coefficient
$N_{z}$	A set of neighboring zones of the zone *z*
*n*	the number of neighboring zones

**Table 2:** Average distance coefficient according to type of neighboring zone.

	$\tau_{A}$	$\tau_{B}$	$\tau_{C}$
Figure	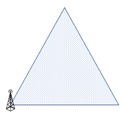	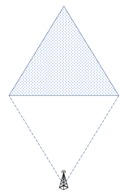	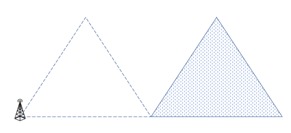
$D_{avg}$	0.608	1.175	1.540
	$\tau_{D}$	$\tau_{E}$	$\tau_{F}$
Figure	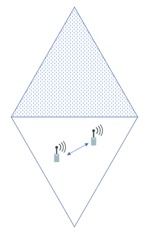	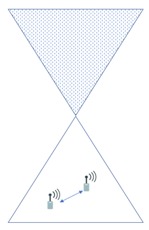	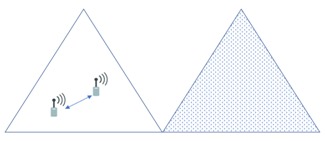
$D_{avg}$	0.706	1.08	1.225

**Table 3:** The number of interferer zones in neighboring zones, *n*.

Mode	Link	$\tau_{A}$	$\tau_{B}$	$\tau_{C}$	Link	$\tau_{D}$	$\tau_{E}$	$\tau_{F}$
*ffr*	*CE*→*CE*	0	1	2	*CE*→*D2D*	0	0	3
*ffr*	*D2D*→*CE*	2	2	4	*D2D*→*D2D*	0	0	3
*ss*	*CE*→*CE*	0	6	12	*CE*→*D2D*	0	0	0
*ss*	*D2D*→*CE*	0	0	0	*D2D*→*D2D*	1	2	4

**Table 4:** Simulation parameters.

Parameter	Value
Macro cell structure	Hexagonal grid 3-tier
	19 cell sites
Center frequency	2.0 GHz
System bandwidth	10 MHz
The number of sensors	12/24/60 of 50% cellular sensor
	50% D2D sensor
Cell radius	R = 866 m
Inter-distance sensors	10 m ≤ distance ≤ 50 m
for D2D link	
Resource allocation	1 Resource block (RB) per a sensor
Noise figure	−147 dBm/Hz
Path loss model:	$23.5\log_{10}\left(distance\left(m\right)\right)+57+23\log_{10}\left(f/5\right)$
Winner II B5f	

## Appendix A. Average Distance

The $\tau_{A}$, $\tau_{B}$ and $\tau_{C}$ are the average distance between a point and an area. It is calculated by integrating distance between the point and a point in the area, and it is divided by the area. To facilitate integration, the area is divided by half and then summed after the integration. The two separated areas, $A_{A}$ and $B_{A}$ for $\tau_{A}$, are derived as

(A1) $$\begin{array}{cc} A_{A} & =\int_{0}^{\frac{1}{2}}\int_{0}^{\sqrt{3}x}r\left(x,y\right)dydx\approx 0.0994, \end{array}$$

(A2) $$\begin{array}{cc} B_{A} & =\int_{\frac{1}{2}}^{1}\int_{0}^{-\sqrt{3}x+\sqrt{3}}r\left(x,y\right)dydx\approx 0.1637, \end{array}$$

(A3) $$\begin{array}{cc} \tau_{A} & =D_{avg}\left(q\right)=\frac{\int_{q}r\left(x,y\right)dS}{S}=\frac{A_{A}+B_{A}}{S}\approx 0.6075, \end{array}$$

where $r\left(x,y\right)=\sqrt{x^{2}+y^{2}}$ is distance function between dS to the eNB, which is at the point (0,0). Similarly, $\tau_{B}$ and $\tau_{C}$ can be described as

(A4) $$\begin{array}{cc} A_{B} & =\int_{-}^{0}\frac{1}{2}\int_{\frac{\sqrt{3}}{2}}^{\sqrt{3}x+\sqrt{3}}rdydx\approx 0.254, \end{array}$$

(A5) $$\begin{array}{cc} B_{B} & =\int_{0}^{\frac{1}{2}}\int_{\frac{\sqrt{3}}{2}}^{-\sqrt{3}x+\sqrt{3}}r\left(x,y\right)dydx\approx 0.254, \end{array}$$

(A6) $$\begin{array}{cc} \tau_{B} & =D_{avg}\left(q\right)=\frac{\int_{q}r\left(x,y\right)dS}{S}=\frac{A_{B}+B_{B}}{S}\approx 1.175, \end{array}$$

(A7) $$\begin{array}{cc} A_{C} & =\int_{1}^{\frac{3}{2}}\int_{0}^{\sqrt{3}x-\sqrt{3}}rdydx\approx 0.298, \end{array}$$

(A8) $$\begin{array}{cc} B_{C} & =\int_{\frac{3}{2}}^{2}\int_{0}^{-\sqrt{3}x+2\sqrt{3}}r\left(x,y\right)dydx\approx 0.369, \end{array}$$

(A9) $$\begin{array}{cc} \tau_{B} & =D_{avg}\left(q\right)=\frac{\int_{q}r\left(x,y\right)dS}{S}=\frac{A_{C}+B_{C}}{S}\approx 1.541. \end{array}$$
